# Insight gained from responses to surveys on reference dosimetry practices

**DOI:** 10.1002/acm2.12081

**Published:** 2017-04-11

**Authors:** Bryan Muir, Wesley Culberson, Stephen Davis, Gwe‐Ya Kim, Yimei Huang, Sung‐Woo Lee, Jessica Lowenstein, Arman Sarfehnia, Jeffrey Siebers, Naresh Tolani

**Affiliations:** ^1^ Measurement Science and Standards National Research Council of Canada Ottawa ON Canada; ^2^ Department of Medical Physics University of Wisconsin Madison WI USA; ^3^ Medical Physics Unit McGill University Montreal QC Canada; ^4^ Department of Radiation Medicine and Applied Sciences UC San Diego School of Medicine La Jolla CA USA; ^5^ Department of Radiation Oncology Henry Ford Health System Detroit MI USA; ^6^ Department of Radiation Oncology University of Maryland School of Medicine Baltimore MD USA; ^7^ Department of Radiation Physics UT M.D. Anderson Cancer Center Houston TX USA; ^8^ Department of Radiation Oncology University of Toronto Toronto ON Canada; ^9^ Department of Radiation Oncology University of Virginia Health System Charlottesville VA USA; ^10^ Department of Radiation Therapy Michael E. DeBakey VA Medical Center Houston TX USA

**Keywords:** best practice, ionization chambers, reference dosimetry

## Abstract

**Purpose:**

To present the results and discuss potential insights gained through surveys on reference dosimetry practices.

**Methods:**

Two surveys were sent to medical physicists to learn about the current state of reference dosimetry practices at radiation oncology clinics worldwide. A short survey designed to maximize response rate was made publicly available and distributed via the AAPM website and a medical physics list server. Another, much more involved survey, was sent to a smaller group of physicists to gain insight on detailed dosimetry practices. The questions were diverse, covering reference dosimetry practices on topics like measurements required for beam quality specification, the actual measurement of absorbed dose and ancillary equipment required like electrometers and environment monitoring measurements.

**Results:**

There were 190 respondents to the short survey and seven respondents to the detailed survey. The diversity of responses indicates nonuniformity in reference dosimetry practices and differences in interpretation of reference dosimetry protocols.

**Conclusions:**

The results of these surveys offer insight on clinical reference dosimetry practices and will be useful in identifying current and future needs for reference dosimetry.

## 1 Introduction

Determination of absorbed dose in external photon and electron beams is realized by following protocols^1^, ^2^ that specify reference conditions and required corrections to the reading of a calibrated reference‐class ionization chamber. The addendum to the TG‐51 protocol^3^ was published in 2011 and includes refinements to the original protocol for high‐energy photon beam dosimetry. These instructions only relate to the measurement of absorbed dose, and therefore do not provide guidance on ancillary equipment or measurements of depth‐dose curves required for beam quality specification. There is also room for interpretation on how to practically implement the reference dosimetry protocols.

Some reports do provide guidelines related to certain aspects of reference dosimetry. The IEC 60731^4^ report provides specifications required for electrometers but these recommendations are rather generous, allowing a relative combined uncertainty of 1.6%. Morgan et al.^5^ describe the more realistic uncertainties that can be achieved in the clinic with modern electrometers at the 0.3% level. The American Association of Physicists in Medicine (AAPM) TG‐106 report^6^ on beam data commissioning provides recommendations on scanning procedures and this is a good starting point for depth‐dose determination. Tailor et al.^7^ and Followill^8^ describe guidelines and common sources of error related to the practical clinical implementation of the TG‐51 protocol.

As part of its charge to review different calibration issues, the AAPM working group on the review and extension of beam quality conversion factors for the TG‐51 protocol (WGTG51) aims to prepare recommendations for best practice regarding procedures, such as depth‐dose acquisition and corrections, and ancillary equipment, such as electrometers, barometers, thermometers and associated calibration, required for reference dosimetry of external radiation therapy beams calibrated following the TG‐51 protocol. To this end, two surveys were prepared on current reference dosimetry practices.

A detailed survey was sent to WGTG51 members (designated here as LD for long detailed survey) and focuses in great detail on reference dosimetry practices followed at their respective institutions. This survey was designed to be descriptive and it would therefore be impossible to administer this survey to a large sample. A weakness with this approach is that it is difficult to draw conclusions with such a small sample size. Therefore, a less detailed, data‐based survey was posted on the AAPM website and on an international medical physics list server. This survey (designated here as SP for short posted survey) was designed to supplement the results from the LD survey and gain insight on current practices but remain short enough to maximize the response rate. This manuscript documents the results of these surveys with the aim of gaining insight into understanding current clinical reference dosimetry practices.

Brand names, model designations, and/or manufacturers are given in this report for identification only, and do not imply recommendation or endorsement by the authors, their affiliated clinics, or the AAPM, nor do they imply that the products are necessarily the best or only instruments available for the purpose. The content of this manuscript is not to be taken as recommendations or guidelines and is not endorsed by the AAPM.

## 2 Methods

**Figure 1:**
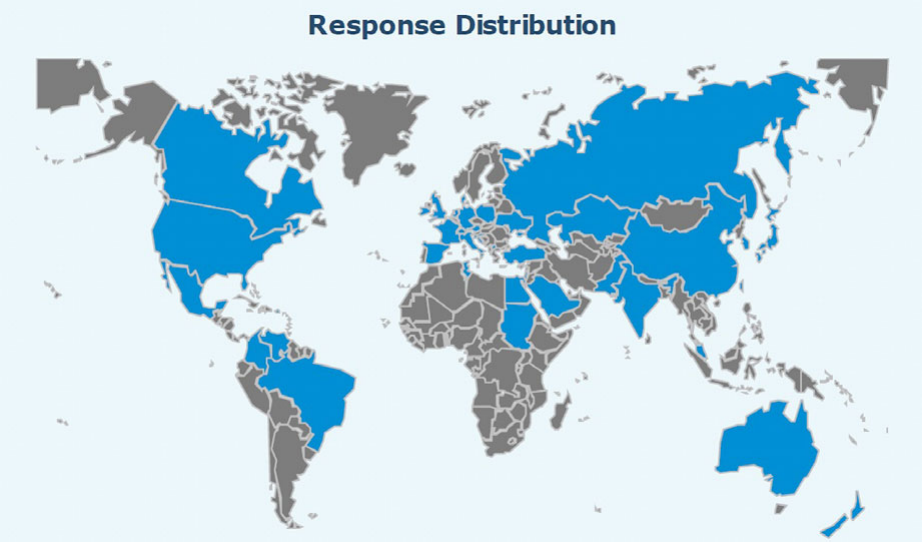
A world map showing the distribution of responses to the short (SP) survey. Countries from which responses came are shaded blue.

As described in the introduction, two surveys were created that deal with reference dosimetry practices. A short data‐based survey (SP) was posted on the AAPM website and medical physics list server. There was no requirement that one must be an AAPM member to complete the survey. In total, 341 respondents started the survey and 190 completed it. Seventy percent of the respondents were from the United States but there were responses from several countries worldwide. Figure 1 shows a world map indicating the response distribution. Around 83% of respondents were AAPM members. A more detailed survey (LD) was sent to seven members of the WGTG51 from various clinics in the United States and Canada.

The topics covered in these surveys include:

1. Beam quality specification: Measurements in water as a function of depth to determine percentage depth‐dose (PDD) curves for beam quality specification to select beam quality conversion factors. Respondents were asked about the choice of detector typically used, how the data were acquired (e.g., resolution), geometric set‐up (the choice of water tank and detector positioning), conversion of detector reading to dose (any corrections to the signal as a function of depth or relative shift of the detector), any other equipment required for measurements as a function of depth (e.g., thermometer), scanning software and whether or not they use lead foil in high‐energy photon beams to account for electron contamination.
2. Absorbed dose under reference conditions: Measurements to determine absolute dose according to codes of practice for reference dosimetry. Respondents were asked about the type of ionization chamber used, how k_Q_ factors were selected, ancillary equipment used (triaxial cable, electrometer, thermometer, barometer) and performance requirements, frequency of calibration of ionization chamber and ancillary equipment, water tank, and geometric set‐ up, if and how tests are performed to determine the integrity of the dosimetry system and ancillary equipment.

These questions covered both electron and photon beam dosimetry measurements. The list of questions will not be provided here for clarity and brevity, rather they will be described along with the results.

## 3 Results

### 3.A Beam quality specification using percent depth‐dose measurements

#### 3.A.1 Scanning water phantom accuracy, resolution, and detectors employed

No questions about the scanning water phantom and associated accuracy were asked in the SP survey. In response to the LD survey, all seven participants used a variety of water tank solutions from IBA Blue Phantom (both BP1 and BP2), Standard Imaging DoseView 3D, as well as the Sun Nuclear Corp. 1D and 3D scanners. The manufacturers accompanying software were used in all instances. All systems have very similar specifications and their positioning accuracy is quoted as 0.1 mm. All participants scan their beam in the vertical direction, although one center also does scan in the horizontal beam orientation.

Of the responses to the SP survey, 96% indicated that a cylindrical ion chamber was used for percentage depth‐dose measurements of photon beams for beam quality specification and 70% indicated that a shift was used to convert the detector reading to dose to water and account for the effective point of measurement. The remaining 4% used plane‐parallel chambers, shielded diodes, MOSFET or diamond detectors for photon beam quality specification.

**Figure 2:**
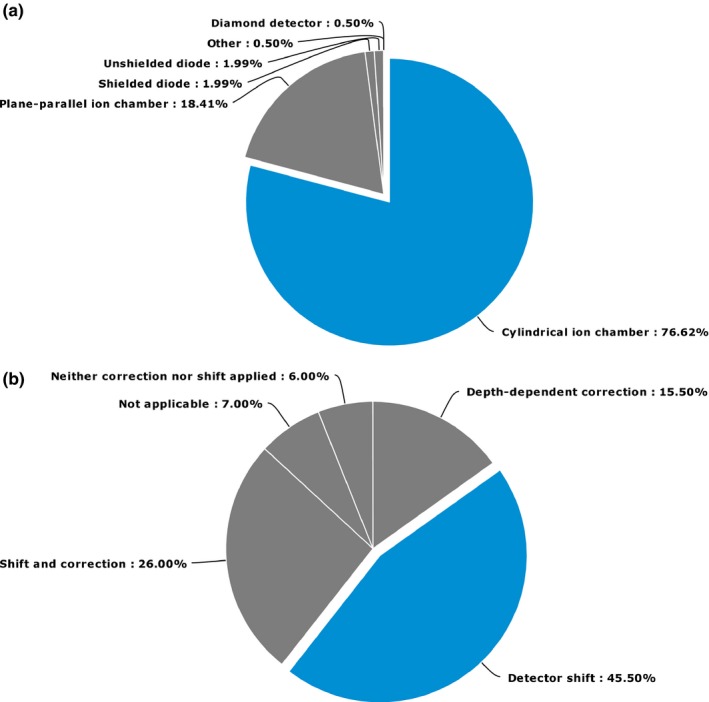
Responses to SP questions about beam quality specification for electron beams. Panel (a) shows the distribution of detectors used for measurements while panel (b) shows how these measurements were corrected.

More variation among the choice of detectors was indicated in responses to the SP survey for electron beam quality specification. Figure 2 a shows the distribution of responses. Cylindrical detectors were the most popular choice (77%) with 18% using plane‐parallel chambers. Few clinics used diodes or diamond detectors in electron beams. In electron beams, 46% used a detector shift (e.g., to account for gradient and/or wall effects) to correct the detector reading, 16% used a depth‐dependent correction while 26% used a combination of a shift and correction as a function of depth (presumably to correct for variation in stopping‐power ratios as recommended by Burns et al.^9^). The remaining respondents did not correct detector readings. Figure 2(b) show the distribution of responses indicating how detector readings are corrected for beam quality specification of electron beams.

Similarly, most (six) of the seven WGTG51 members given the LD survey used a cylindrical scanning chamber for photon PDD measurement, while one used a photon field diode. As with the SP survey, more variation was observed for electron PDD measurement. Two institutions used a diode, one used a combination of diode and chamber, and four used cylindrical scanning chambers. When an ionization chamber is used, the effective point of measurement was taken as 0.6 × r_cav,_ where r_cav_ is the chamber radius, upstream of the central axis of the chamber in photon scanning, and 0.5×r_cav_ in electron scanning. When an ionization chamber is used for scanning, the majority of respondents stated that the ionization chamber is shifted upsteam by the scanning program before taking measurements, while two institutions applied the chamber shift manually after the data were acquired.

The actual operating resolution and reproducibility of the scanning system was not directly verified annually by any of the WGTG51 LD survey participants. In some cases only the mechanical arm positioning was checked against a ruler for accuracy and reproducibility. The periodicity of this check was set by the clinics.

Both continuous scans as well as step‐by‐step mode of scanning were used by WGTG51 members who responded to the LD survey. The choice was a balance between time required to take measurements and the accuracy and noise level of the scans taken. To reach a good balance, one center chose to do step‐by‐step mode with a depth dependent scan resolution (setting 0.5–1 mm below dmax, while increasing this to 2–3 mm past dmax), while the rest used continuous scanning with roughly a mean of 0.7 mm scan resolution. It was noted that the profiles are noise limited when step sizes smaller than 0.5 mm is used.

#### 3.A.2 Water phantom environmental stability and set‐up

No questions related to environmental monitoring for depth‐dose measurements or the scanning phantom set‐up were asked in the SP survey.

All answers to the LD survey indicate that the reservoir or water tank was either stored in the room or left in the room overnight before scanning so the temperature of the water would be at equilibrium. Of the seven physicists polled, six did not monitor temperature stability and one measured temperature every two to five hours during scanning measurements and therefore did not monitor the stability of the water temperature over the course of a scan. One respondent noted that water temperature and/or atmospheric pressure does not change significantly over the course of one scan (typically five minutes).

Of those given the LD survey, the SSD was set using room lasers, optical distance indicators, and calibrated pointers as well as a combination of these methods.

All physicists polled with the LD survey used profiles at different depths (CAX search) for detector positioning and ensuring vertical movement along the beam axis.

All respondents to the LD survey still used lead foil when making PDD measurements for high‐energy photon beams.

### 3.B Measurements required for absorbed dose determination

#### 3.B.1 Ion chamber used for dose determination

**Figure 3:**
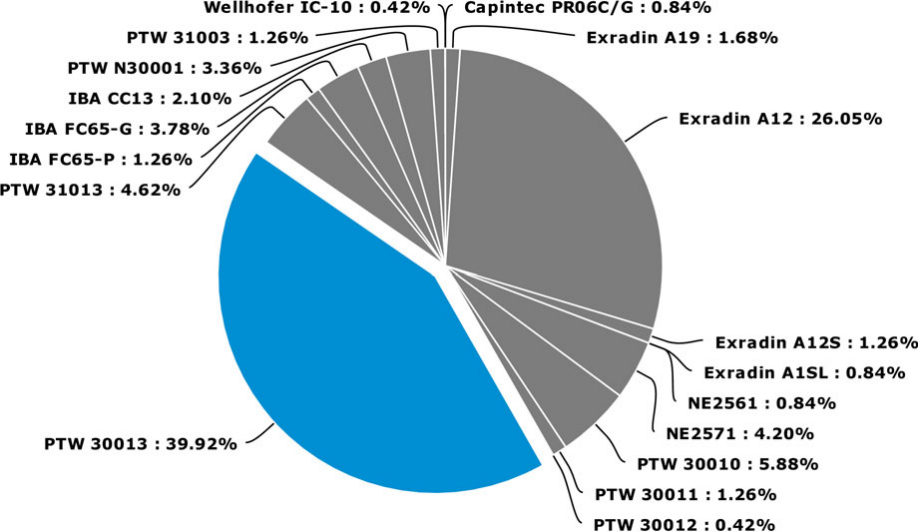
The distribution of responses to the SP survey indicating the ion chamber type used for photon beam reference dosimetry.

The SP survey asked what chamber is most commonly used for reference dosimetry of photon beams and all respondents indicated cylindrical chambers, with the PTW 30013 and Exradin A12 chambers being the most popular choices. Figure 3 shows the distribution of chambers used for reference dosimetry of photon beams. Of these, 82% indicated that the same chamber was used for reference dosimetry of both photon and electron beams. Those that did not use the same chamber for photon and electron dosimetry typically used plane‐parallel chambers for electron beam calibrations, with the most popular choices being PTW Roos and IBA NACP‐02 chambers, although a variety of other plane‐parallel chambers are also used. Of those using plane‐parallel chamber, 56% cross‐calibrated these chambers against stable cylindrical chambers as detailed in the TG‐51 protocol.

For photon beam dosimetry, five of the seven institutions given the LD survey used an Exradin A12 chamber while the others used either an NE2571 or a PTW30013.

Three out of the seven respondents to the LD survey used parallel‐plate chambers for some or all of their electron beam reference dosimetry. One institution used the PTW Markus chamber for all electron energies. Another used a PTW Roos chamber only for their 6 MeV total skin irradiation beam. Another used the Exradin P11 for 4 MeV electrons. All three of these institutions performed cross‐calibration of these parallel‐plate chambers against cylindrical reference‐class chambers.

#### 3.B.2 Ion chamber stability monitoring

Both SP and LD surveys had questions about how often reference chamber calibration was performed and this is typically either annually or every second year, by obtaining a calibration coefficient factor for the clinics primary ionization chamber from a secondary standards lab such as ADCL or its equivalent. However, 4% of respondents to the SP survey indicated that the period between calibrations is greater than 2.5 yr.

TG‐51 and its associated addendum require redundant checks of reference dosimetry systems. There are several options for monitoring N_D,w_ drifts including having independent dosimetry systems, using a radiation check source or cobalt‐60 irradiator or using clinical radiation sources such as ^137^Cs (GYN implant source) or ^60^Co (Gamma Knife) with a reproducible chamber holder to routinely check stability. The LD survey asked about N_D,w_ drifts, which are monitored by all participants with a tolerance of 0.5%. Two of the institutions used separate chambers for TG‐51 calibration, which were cross‐calibrated against the local standard annually using 60 Co or 6 MV photon beams. One institution cross‐checked reference chambers twice per year in addition to the ADCL calibration. One center performed a CT scan of the chamber upon purchase to ensure lack of obvious defects.

#### 3.B.3 k_Q_ selection

All but one clinic given the LD survey avoided changing their k_Q_ factor from year to year, but rather measured and possibly tuned the beam to ensure beam quality is preserved and consistent with commissioning data and/or data present in the treatment planning system. The acceptable beam quality tolerances beyond which beam tuning is required vary among institutions, but they range from a tolerance of 0.001 difference in k_Q,_ to a match of the PDD against the commissioning data set (within 2%/2 mm at one center, or within 1% at another center, etc.).

#### 3.B.4 Water phantom and set‐up for beam calibration

Questions about water phantom set‐up were not asked in the SP survey. The LD survey asked questions on the choice of water phantom and set‐up for reference dosimetry measurements. Only one of seven institutions used an in‐house water phantom while the other institutions used commercial phantoms for beam calibration. In particular, this institution used two separate phantoms for photons and electrons, with the photon calibrations at a fixed depth of 10 cm, and 0.01 mm incremental positioning for the electron phantom with an overall accuracy of about 0.1 mm. Overall, mechanical resolutions of the phantoms for all respondents were within 0.1 mm.

The responses to the LD survey indicate that the accuracy and reproducibility of ion chamber positioning was verified with different methods depending on the clinic. One physicist noted that as a quick sanity check, the depth for photon beam measurement at 10 cm was verified using a ruler. Positioning accuracy can be checked by monitoring the match (and possible drift) of the projected shadow of the ionization chamber, as the chamber is moved to depth in water, relative to the projected cross‐hair at the bottom of the water tank. One institution performed electron measurement separately and stated a relative phantom accuracy maintained to about 0.01 mm. Most of the reporting institutions used a water‐ proof chamber, though two institutions reported that they had sleeves that accommodated non‐waterproof chambers.

Regarding the specifications of the 3‐D water tank system, values vary between manufacturers. The respondents to the LD survey reported a range of resolution specifications from 0.01 mm to 0.5 mm.

The LD survey also asked that each institution report the frequency of quality assurance performed on the water tank positioning system for absorbed dose measurements. The respondents had different methods and frequencies for performing a check of the system positioning accuracy. One respondent used a ruler. Another respondent checked with a visual inspection of the shadow of the chamber. Yet another user reported using a micron stage to check alignment. Lastly, one respondent only checked the positioning accuracy when there was a significant drift in the machine output (presumably to eliminate the scanning system from being the culprit).

### 3.C Ancillary equipment for absorbed dose determination

#### 3.C.1 Electrometer used for TG‐51 calibration

Respondents to both SP and LD surveys were asked about their electrometer make and model. The LD survey asked for the minimum and optimal specifications for readout to be used for a TG‐51 calibration.

**Figure 4:**
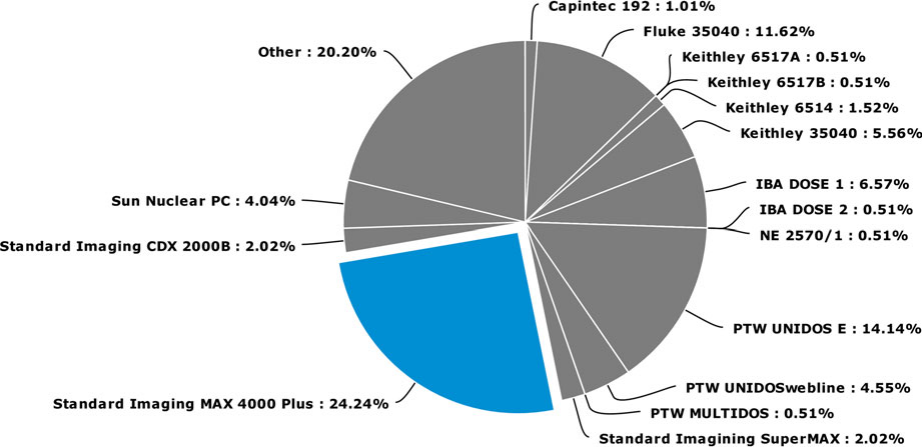
The distribution of responses to the SP survey indicating the electrometer type used for reference dosimetry.

Both the SP and LD survey responses indicated that a wide variety of electrometer makes and models were used. In fact, not one type was used by more than 25% of respondents. Figure 4 shows the distribution of responses to the SP survey on electrometer type used.

The electrometer models used typically have the ability to set bias on the chambers using an internal power supply.

All electrometers used have nominal specifications of accuracy and precision to be suitable for use with a reference class ionization chamber for the AAPM TG51 protocol.

The LD survey asked respondents to specify minimum and optimal specifications required before an electrometer could be considered for TG‐51 and these varied among institutions. Some replied with a minimum requirement for the electrometer resolution such as absolute current values (e.g., 0.01 pA), charge values (e.g., 0.0001 nC), or relative standard deviations of charge or current readings in percent (e.g., 0.1%). In general, there was no consensus in the polling results on the specifications required, which is understandable since the detailed specifications of an electrometer are not prescribed in the TG51 protocol.

Responses to the SP survey indicated that 82% of physicists had their chamber‐ electrometer system calibrated together and that the calibration interval was typically two years.

One repondent to the LD survey noted that the leakage requirements of their electrometer was less than 100 fA.

Lastly, one respondent to the LD survey noted that the calibration coefficient for the electrometer should be stable and within 0.1% of unity.

#### 3.C.2 Dosimetry system cabling, leakage and settling

In response to a question on the LD survey regarding cable noise, capacitance, or system integrity checks, no center explicitly monitored cable noise, capacitance, or performed explicit integrity checks prior to the TG‐51 measurement. However, some centers noted that they considered cabling as a potential culprit when leakage or noise was greater than expected. Also noted was the fact that clinically relevant problems would show up as large differences in linac output compared to what was expected. Some of the centers performed annual cross calibrations of their TG‐51 ion chambers against the local standard, which would be a check on the system.

There was a wide variety of responses to the LD survey regarding the amount of time the system should be on prior to starting measurements. In one center the electrometer was always left on, while the rest of the respondents switched on the electrometer 5 and 30 min prior to making measurements. Four of the seven centers considered system leakage before starting measurements, and this ranged from less than 150 fA to less than 0.5% of the chamber reading, which could be about 10 pA. The same acceptable leakage range was noted after making measurements. One of the seven centers used separate triaxial cables for routine QA measurements and TG‐51 calibration.

#### 3.C.3 Temperature measurement for beam calibration

Both SP and LD surveys asked about the type of thermometer used for the TG‐51 calibration procedure. From the SP survey, 62% of respondents used a digital thermometer while 18% and 16% used mercury and alcohol thermometers, respectively. The SP survey also asked about whether these thermometers had traceable calibrations (58% did) and if they were recalibrated (48% indicated that they never recalibrated their thermometer).

All respondents to the LD survey used either digital (5) or alcohol (2) thermometers. Specifications were given in terms of either resolution or accuracy. The resolution of the thermometers was typically 0.1°C (though one clinic indicates 0.05°C) and accuracy was quoted as 0.2°C by two clinics.

One respondent to the LD survey indicated that they had their thermometer calibrated once per year but all others did not recalibrate their instruments. Instead, most users performed cross‐checks against other thermometers and investigated abnormalities. One respondent said that they verified the zero point of the thermometer in ice water when purchased. Most respondents said that they ensured that the thermometer was not touching the side wall of the phantom where there may be a temperature gradient and was at roughly the same depth as the detector.

#### 3.C.4 Pressure measurement for beam calibration

The SP survey asked about the type of barometers used for pressure measurements for TG‐51 calibrations. Digital barometers were used by 51% while 28% used aneroid and 19% used mercury barometers. Of these respondents, 55% said that their barometer had a calibration traceable to NIST, but 43% indicated that they never recalibrated the instrument.

Aneroid, mercury or digital barometers were used for pressure measurement by respondents to the LD survey. Three respondents indicated that the resolution of their barometer is 0.1 mmHg, while one indicated 0.1 kPa (a factor of almost 10 smaller). Only one respondent stated a specification on accuracy of their barometer, and it was better than 1.5 mmHg (0.2 kPa). One of the respondents purchased a new NIST traceable digital barometer every 2 to 3 yr and cross‐check all other barometers with that device. None of the other respondents surveyed calibrate their barometer, although one cross‐checked two instruments and three checked their barometers against local weather stations.

Only 5% of respondents to the SP survey measured relative humidity before TG‐51 calibrations. No respondents to the LD survey said that they checked humidity before performing TG‐51 measurements.

## 4 Discussion

It is interesting that, although the TG‐51 protocol specifies that only ion chambers be used for beam quality measurements, some physicists are using other detector types. Although the protocol does not specifically prohibit the use of other detectors, there is not mention of the option to use them and the entire discussion on beam quality specification is based on the use of ion chambers.

Questions about detectors used for beam quality specification indicated that most users made depth‐ionization measurements with a cylindrical chamber in photon beams and corrected the reading with a shift of the detectors point of measurement. This is likely appropriate considering the lack of variation in stopping‐power ratios in photon beams.

The respondents to the SP survey indicated that both cylindrical and plane‐parallel chambers are being used for electron beam quality measurements. The results indicate that 46% only apply a detector shift, even though the variation in stopping‐power ratio with depth is well known and significant.^9^

Responses to the LD survey indicated that the EPOM shifts of 0.6 × r_cav_ for photon beam quality measurements and 0.5 × r_cav_ for electron beam quality measurements recommended in TG‐51 using cylindrical chambers are still being used. However, recent publications suggest that these shifts are not correct.^10^, ^11^, ^12^

Responses to the LD survey indicated that little and varied scanning tank testing is performed although TG‐106 recommends testing the positioning accuracy/reproducibility of the tank upon purchase and then yearly preventative maintenance of the system.

Most respondents to the LD survey indicated that temperature and pressure were not monitored during water scanning for beam quality measurements with the assumption that little variation occurs over the course of a scan. Because of the high heat capacity of water it is very unlikely that temperature variability will affect these measurements. However, atmospheric pressure can be more variable especially during a storm. A change in pressure of 0.1 kPa over the course of a scan would introduce an error of 0.1% if not accounted for using the P_tp_ correction. Another consideration for pressure variation is that if one normalizes the results to an external monitor chamber (i.e., a field or transmission chamber), the component of P_tp_ related to pressure can be ignored and variations in pressure introduce no bias as long as both chambers communicate with the atmosphere.

Responses to the LD survey indicated that various methods are used to set SSD for both beam quality measurements and absorbed dose determination. The addendum to TG‐51 suggests that the use of a calibrated pointer is preferable for an SSD set‐up as the accuracy acheivable with this method is 0.2 mm.^3^ For depth‐ionization measurements as long as the SSD is close to 1 m then uncertainty in scans from SSD setting is only related to small variations in field size, which should be negligible compared to real field size variations. However, there will be an error in absolute dose determination if SSD setting is inaccurate. If the SSD is off by 0.2 mm, the error in absolute dose measurement is less than 0.05% but if SSD is off by 1 mm the error in absolute dose is 0.2%.

Responses to the LD survey indicated that beam profiles are used for detector positioning for scanning. This method may lead to improved accuracy in positioning since physical indicators (markings on phantom, crosshairs) may not be truly aligned with the beam axis.

Respondents to the LD survey indicate that they still use lead foil for high‐energy photon beam quality measurements. Note that the addendum to the TG‐51 protocol^3^ states that the simplified procedure without the use of lead foil can be used as the default method for some beams to avoid operational errors since the use of lead foil only introduces an error in k_Q_ of 0.2%.^7^

A variety of ion chambers are used for both photon and electron beam absorbed dose determination. However, the choice of chamber is unimportant if that chamber has not been shown to be adequate for reference dosimetry. The addendum to the AAPM's TG‐51 protocol details specifications of a reference‐class ion chamber (at least for MV photon beam dosimetry) and strongly recommends that any chamber used for reference dosimetry be well characterized.

Various responses were observed regarding the accuracy and resolution of scanning tank systems and water phantoms used for absorbed dose measurements. For a TG‐51 type measurement, localizing the geometric center of the chamber is very important, considering steep depth‐dose gradients. The specifications of water tank systems are more involved than any single number. The user will want to know the uncertainty of the position of the chamber after an origin has been set in the water tank software. For a TG‐51 type calibration, the user may elect to set origin with the chamber geometrically bisecting the surface of water using mirror symmetry. The chamber travel is then indexed along a single axis of motion (parallel to the beam) to the depth of calibration. Beam quality scans are performed in addition to the static measurement location, so positioning uncertainties will play a role in the calculation of the beam quality specifier as well as the location of the chamber for the calibration reference point. With all of this in mind, the addendum to the TG‐51 protocol indicates that 0.33 mm is appropriate and achievable for a TG‐51 calibration. Positioning uncertainties at 0.5–1 mm, as one of the respondents to the LD survey answered, begins to encroach on the level of accuracy for the calibration resulting in an uncertainty in absorbed dose greater than 0.25%.

Many survey responses indicated that the chamber/electrometer system is calibrated together every 2 yr or that the electrometer is calibrated separately but still bi‐annually. There are no formal recommendations from the AAPM for the calibration frequency of electrometers, but a bi‐annual calibration would seem appropriate since the ion chamber and electrometer work as a system and need to be checked at similar intervals.

Various responses about the leakage of the chamber/electrometer system were observed. The amount of permissible leakage should be analyzed as a fraction of the total current measured when connected to an ion chamber and irradiated in the measurement position. Typically, for a well‐behaved chamber‐electrometer system this is less than 50 fA and much less than 0.1% of the reading under irradiation.^3^ Leakage should be kept small since the offset on the net current due to leakage will relate directly to the bias on the overall measurement of dose.

One respondent to the LD survey indicated that the value of their electrometer calibration coefficient should be stable and within 0.1% of unity. Although the absolute value of the electrometer calibration need not be unity or within 0.1% of unity, it is important that the value is stable and properly characterized by an ADCL before initial use. Analyzing the variation of the calibration coefficient over time will give an indication of the stability of the electrometer. Any problems or instabilities should be communicated to the manufacturer, since it will directly affect the measurement of dose.

The accuracy of thermometers used for TG‐51 measurements was quoted as 0.2°C by two respondents to the LD survey. If this accuracy is a true estimate of uncertainty then it would lead to an uncertainty in the measurement of dose to water of 0.07%.

One respondent to the LD survey indicated that accuracy of their barometer was better than 1.5 mmHg (0.2 kPa). Taken as an estimate of uncertainty in pressure measurement this leads to an uncertainty in dose measurement of 0.2%.

Very few physicists polled monitor humidity and this is likely acceptable as long as relative humidity is in the range 10–90%, over which the correction for effects of humidity varies by less than 0.15%.^13^

## 5 Conclusions

This study documents the results of two surveys on reference dosimetry practices initiated by the AAPM working group on the review and extension of beam quality conversion factors for the TG‐51 protocol (WGTG51). From the responses to these surveys some interesting insights on reference dosimetry practices are obtained.

Given recent research on ion chamber shifts for beam quality specification measurements, recommendations could be implemented to improve the accuracy of these measurements. The survey results observed here indicate that very few clinics have implemented updated shifts.

Respondents to the LD survey performed no environmental monitoring while measuring depth‐ionization scans for beam quality specification. However, this will likely have little impact on dosimetry measurements because variations in environmental conditions are typically small over the short time period required for a scan.

For absolute dosimetry measurements, 82% of respondents to the SP survey used the same cylindrical chambers for photon and electron beam calibrations. This indicates that physicists are comfortable using cylindrical chambers for reference dosimetry of electron beams despite the recommendations in the TG‐51 and TRS‐398 protocols that plane‐parallel chambers be used for electron beams with energies less than 10 MeV (or are not using electron beams with energies less than 10 MeV). This, combined with recent publications^11^, ^12^ that point out the suitability of cylindrical chambers in low‐energy electron beams, will likely have an impact on recommendations in future dosimetry protocols.

Only three out of seven of the respondents to the LD survey indicated that they monitored the stability of their dosimetry system by performing cross‐checks with redundant systems (aside from tracking ADCL N_D,w_ drifts) despite the fact that TG‐51 specifically states that a redundant system must be in place for reference dosimetry measurements. Monitoring chamber stability, specifically before and after ADCL calibration to ensure no damage occurred during shipping, is an important part of the clinical medical physicists reference dosimetry program.

For environmental monitoring measurements required for absorbed dose determination, only 55–58% of respondents to the SP survey used NIST traceable equipment for temperature and pressure measurement. Of these, 43–48% never recalibrated these instruments. However, from the LD survey results, a cross‐check of several instruments was typically performed to assess performance of thermometers and barometers. This is likely acceptable since the readings of several instruments are not likely to drift in the same way over a given period of time.

The results of these surveys will prove useful in that they offer valuable insight on current reference dosimetry practices and will help provide guidance in future updated protocols for reference dosimetry.

## Conflict of interest

None of the authors have a conflict of interest related to this manuscript.
